# A Cross-Sectional Randomised Study of Fracture Risk in People with HIV Infection in the Probono 1 Study

**DOI:** 10.1371/journal.pone.0078048

**Published:** 2013-10-29

**Authors:** Barry S. Peters, Melissa Perry, Anthony S. Wierzbicki, Lisa E. Wolber, Glen M. Blake, Nishma Patel, Richard Hoile, Alastair Duncan, Ranjababu Kulasegaram, Frances M. K. Williams

**Affiliations:** 1 Department of Infectious Diseases, Kings College London, London, United Kingdom; 2 Honorary Consultant Physician, Guys and St Thomas’ Foundation Hospitals, London, United Kingdom; 3 Department of HIV and GU Medicine Physician, Guys and St Thomas’ Foundation Hospitals, London, United Kingdom; 4 Department of Chemical Pathology, Guys and St Thomas’ Foundation Hospitals, London, United Kingdom; 5 Twins Research Unit, Department of Rheumatology, Kings College London, London, United Kingdom; 6 Department of Nuclear Medicine, Kings College London, London, United Kingdom; 7 School of Medicine, Kings College London, London, United Kingdom; 8 Department of Dietetics, Guys and St Thomas’ Foundation Hospitals, London, United Kingdom; 9 Department of HIV and GU Medicine, Guys and St Thomas’ NHS Trust, London, United Kingdom; National Institute of Allergy and Infectious Diseases, United States of America

## Abstract

**Objective:**

To determine comparative fracture risk in HIV patients compared with uninfected controls.

**Design:**

A randomised cross-sectional study assessing bone mineral density (BMD), fracture history and risk factors in the 2 groups.

**Setting:**

Hospital Outpatients.

**Subbbjects:**

222 HIV infected patients and an equal number of age-matched controls. Assessments: Fracture risk factors were assessed and biochemical, endocrine and bone markers measured. BMD was assessed at hip and spine. 10-year fracture probability (FRAX) and remaining lifetime fracture probability (RFLP) were calculated.

**Main Outcome Measures:**

BMD, and history of fractures.

**Results:**

Reported fractures occurred more frequently in HIV than controls, (45 vs. 16; 20.3 vs. 7%; OR=3.27; p=0.0001), and unsurprisingly in this age range, non-fragility fractures in men substantially contributed to this increase. Osteoporosis was more prevalent in patients with HIV (17.6% vs. 3.6%, p<0.0001). BMD was most reduced, and predicted fracture rates most increased, at the spine. Low BMD was associated with antiretroviral therapy (ART), low body mass index and PTH. 10-year FRAX risk was <5% for all groups. RLFP was greater in patients with HIV (OR=1.22; p=0.003) and increased with ART (2.4 vs. 1.50; OR= 1.50; p=0.03).

**Conclusions:**

The increased fracture rate in HIV patients in our relatively youthful population is partly driven by fractures, including non-fragility fractures, in men. Nonetheless, these findings may herald a rise in osteoporotic fractures in HIV patients. An appropriate screening and management response is required to assess these risks and identify associated lifestyle factors that are also associated with other conditions such as cardiovascular disease and diabetes.

## Introduction

Effective anti-viral therapy has markedly reduced mortality from classical AIDS associated diseases. In contrast, non-classical chronic diseases associated with HIV infection such as coronary heart disease and diabetes are increasing in incidence as patients live longer [1–3]. This is compounded by an increase in life-expectancy in the developed world that is shifting the patterns of age-related disease. Hip fracture already constitutes a major cause of rising morbidity in this setting and the incidence has increased by 25% from 1990 to 2000[4] and is projected to increase by 300% by 2050 by the World Health Organisation[5,6].

There is a high incidence of osteoporosis in most HIV cohorts compared to the general population[7]. This may be explained by an increased incidence of risk factors for osteoporosis in people living with HIV [8], direct effects of HIV including immune activation[8–10], the effects of HIV therapy on osteoclast and osteoblast activity[8,11] or through indirect mechanisms such as the effect on vitamin D metabolism, and the association of HIV disease with insulin resistance and lipodystrophy[12]. HAART has been found to be associated with a reduced bone mineral density (BMD) in most studies, but others have not found a significant effect. Most antiretroviral drugs have the potential to interfere with bone metabolism but some drugs, or drug classes have been associated with greater BMD loss than others, notably the protease inhibitor class and tenofovir. A meta-analysis of 12 studies showed a 1.6 times greater loss in BMD for those on a PI regimen compared to those on HAART without a PI [7]. Tenofovir, a nucleoside reverse transcriptase inhibitor, has been associated with reduced BMD in people with HIV, possibly through proximal tubule toxicity[13]. An increased incidence in fractures has already been reported in people with HIV, compared to uninfected cohorts [14,15] and may herald a future trend. Our study addresses the relative contribution of risk factors for osteoporotic fractures present in a large ethically mixed cohort of male and female patients with HIV infection. This will help inform an evidence-based approach to monitoring, screening and management of fragility fractures in this chronic infectious disease.

## Materials and Methods

The protocol was approved by The Bromley PCT Research Ethics Committee (08 H080556). Full informed written consent was obtained.

### Study design and participants

A cross-sectional study of HIV infected patients and age-matched controls was performed. All >3000 patients who regularly attended the HIV outpatient clinic at Guy’s and St. Thomas’ Hospitals, London, UK between January 2009 and April 2010 were eligible providing they were not pregnant and able to give written informed consent. Volunteers were divided into age bands, from 30-34 years, 35-39, 40-44, 45-50, 50 to 54 and ≥55 years of age, with a recruitment target of 20 to 24 in each group. Patients were randomly recruited from general clinics, but not from specialist clinics, to avoid selection bias. The protocol was approved by The Bromley PCT Research Ethics Committee (08 H080556) and is registered with ClinicalTrials.gov Identifier: NCT01669954.

Controls were recruited from the Twins-UK register at the Department of Twin Research (www.twinsuk.ac.uk), King’s College London. This register has >6,000 twin pairs (monozygotic or dizygotic) who are studied regularly for health-related parameters, including bone health. One individual from each twin pair whose visits had been broadly contemporaneous with the HIV infected patients was selected. The twin controls had been recruited from around the United Kingdom, and had DXA scanning between 1998-2000 as part of their assessment. Twins are comparable to singletons for a variety of health and lifestyle traits[16]. It was recognised that it would be impossible to match for ethnicity, as there were very few non-Caucasian volunteers within TwinsUK. There were many fewer males in TwinsUK, but it was possible to select an age-matched group of males (20% of the control group) who had had dual energy X-ray absorbtiometry (DXA) scans and relevant biochemistry. Bone biochemistry and DXA scan, were a routine part of the twin cohort measurements, but testosterone, sex hormone binding globulin (SHBG) and vitamin D levels were not routinely measured among the control group.

### Assessments

Questionnaires were completed by cases and controls and included the risks for fragility fracture, and HIV associated history where appropriate. The fields captured patient characteristics (age, sex, ethnicity and duration of HIV infection), previous fractures, smoking, clinical features of malabsorption, alcohol consumption, chronic diseases associated with secondary osteoporosis (e.g. diabetes, liver disease), body mass index, physical activity index, medication history including anti-retroviral drug therapies, current and nadir CD4, HIV RNA viral load and AIDS defining illnesses. Biochemical assessments included serum calcium, (corrected for albumin of 40g/L), phosphate, 25-OH cholecalciferol (vitamin D), alkaline phosphatase, albumin, sex hormone binding globulin (SHBG), testosterone level, and urine protein:creatinine ratio. A DXA scan of lumbar spine and hip was performed, and the BMD and T scores recorded. Patients and controls were scanned on Hologic DXA machines (Hologic Inc., Bedford, MA) that were cross-calibrated in-vivo by scanning 20 volunteers on both machines. Spine T-scores were calculated using the manufacturer’s US spine reference range[17] and hip scores using the NHANES III range[18]. WHO criteria were used to classify osteoporosis (hip or spine T score, -2.5 or less, i.e. 2.5 SDs below the mean bone mineral density (BMD) value for an individual with the same age, sex and race) or osteopenia (hip or spine T score, between -1 and -2.5). 

The 10 year risk of fragility fracture was calculated using FRAX [19], which integrates clinical risk factors (age, sex, weight, height, previous fracture, parent hip fracture, current smoking, current glucocorticoid use, secondary osteoporosis, and alcohol use (≥3 units/day) to produce a score with or without BMD for an individual by geographic setting. Adjustments do not exist for HIV infection. We used the country specific (UK) tool which, unlike US FRAX, makes no allowance for ethnicity. The outputs are 10-year probability of hip fracture and the 10-year probability of a major osteoporotic fracture (spine, forearm, hip or shoulder fracture)[20]. FRAX has not been validated for people under 40 years old, hence for all younger subjects 40 years was used. 

 The RLFP (remaining lifetime fracture probability) is a Food and Drug Administration (USA) approved tool designed to calculate the cumulative risk of fracture during an individual's remaining lifetime [21,22] A web-based version that utilises multiple decrement life table analysis was applied[23]. Life-expectancy is determined for each subject from which a modified Kaplan-Meier curve is constructed. Thus, the residual lifetime risk of fracture for a 60-yr-old woman is simply the cumulative incidence of fracture (denoted by I_60_) over *T* years, ***I*=*Σh*_*t*_*S*_*t-1*_**, where *h*_t_ is the conditional probability of sustaining a fracture at age *t* years given survival beyond age *t* - 1 years, *S*_t-1_ is the probability of survival beyond age *t* - 1 years free of fracture, and *h*_t_ *S*_t-1_ is the unconditional probability of fracture at age *t* years. As a single time-point DXA scan is used, an assumption is made that the average bone loss is 1.5% of total mineral bone mass per year. The RLFP is based on the life expectancy of the general population (based on sex and current age), is adjusted for major ethic groups but is based on USA data and has no country specific fields. No adjustment is available for life-expectancy for HIV. No adjustment was made for length of time on HAART, but an analysis of BMD and duration of HAART was made.

### Statistical analysis

Data were analysed using STATA (version 11, Stata Corporation, Austin, Texas, USA). Variables were transformed to approximate normal distributions where necessary (Table 1). Comparisons between cases and controls were made using student t test and ANOVA for continuous traits, and Fisher’s exact and chi squared tests for ordinal traits. Multivariate regression analysis was used to estimate the crude and adjusted associations between demographics, clinical parameters and low BMD. All the variables in Table 1 were included in the univariate analysis. All statistical tests were two-sided; associations with *P*-value <0.05 in univariate analyses were considered to be statistically significant and taken forward into multivariate models. In addition, we compared the 10-year probability of hip fracture and the 10-year probability of a major osteoporotic fracture score with and without BMD in each group by the Mann-Whitney test.

**Table 1:** Comparison of fracture risk factors for cases (HIV) and controls (Twins).

	Males			Females		
	Cases	Controls	p-value	Cases	Controls	p-value
N	133	44	-	89	178	-
Age mean (SD)	46.2 (9.4)	46.9 (11.1)	0.69	44.6 (9.0)	45.2 (8.7)	0.63
BMI mean (SD)	24.3 (3.4)	26.9 (3.6)	<0.001	27.9 (5.8)	25.1 (4.4)	<0.001
Parent Fractured Hip (Yes/No)	11/119 (8.5%)	1/43 (2.3%)	0.30	2/87 (2.2 %)	8/170 (10.3 %)	0.50
Smoking (yes/no)	47/86 (35.3%)	10/34 (22.7%)	0.12	11/78 (12.4%)	35/143 (19.7%)	0.14
Alcohol (≥ 3 units/day) (yes/no)	24/109 (18.0%)	0/44 (0%)	0.001	1/88 (1.1%)	2/176 (1.1%)	1.000
Vitamin D (nmol/L) mean (SD)	47.8 (27.3)	^1^	-	37.9 (25.8)	59.8 (33.9)	<0.001
Vitamin D (nmol/L) IQR	28-61	^1^	-	21-46	-	-
Vitamin D (nmol/L) median	43	^1^	-	32	-	-
Corrected Calcium mmol/L-mean (SD)	2.23 (0.08)	2.27 (0 .07)	0.01	2.22 (0.10)	2.33 (0.09)	<0.001
Testosterone (nmol/L) mean (SD)	21.4 (21.5)	^2^	-	0.72 (1.57)	1.12 (0.35)	0.02
Testosterone (nmol/L) IQR	14-21.4	^2^	-	0.4-0.7	-	-
Testosterone (nmol/L) median	19.5	^2^	-	0.4	-	-
PTH (ng/L) mean (SD)	41.5 (22.1)	^2^	-	69.7 (103.1)	38.7 (16.5)	
PTH (ng/L) IQR	28-49	^2^	-	41-64.5	-	-
PTH (ng/L) median	37	^2^	-	49.5	-	-
Phosphate (mmol/L) mean (SD)	1.03 (0.18)	1.01 (0.14)	0.35	1.05 (0.19)	1.09 0.14	0.04
Alkaline phosphatase (IU/L) mean (SD)	89 (34.0)	74 (20)	<0.001	95.7 (52.2)	69.8 (21.8)	<0.001
Alkaline phosphatase (IU/L) IQR	-	-	-	64-110	-	-
Alkaline phosphatase (IU/L) median	-	-	-	69	-	-
SHBG (nmol/L) mean (SD)	56 (27)	-	-	92.2 (45.9)	47.3 (25.5)	<0.001
SHBG (nmol/L) IQR	-	-	-	58-113	-	-
SHBG (nmol/L) median	-	-	-	83	-	-
Osteoporosis(Normal/ Osteopenia/ Osteoporosis)	48/66/19	21/17/6	0.38	32/37/20	129/47/2	<0.001
Spine Hologic BMD mean (SD)	0.99 (0.13)	0.97 (0.16)	0.46	1.00 (0.16)	1.041 (0.12)	0.03
Hip Hologic BMD mean (SD)	0.96 (0.13)	1.03 (0.15)	0.002	0.97 (0.14)	0.96 (0.13)	0.90
Femoral Neck BMD mean (SD)	0.82 (0.13)	0.86 (0.13)	0.09	0.84 (0.15)	0.84 (0.13)	0.80
Fracture Fragility (Yes/No)	6/127 (4.5%)	0/44 (0%)	0.34	0/89 (0%)	5/173 (2.8%)	0.17
Fracture Others (Yes/No)	32/101 (24.1%)	3/41 (6.8%)	0.02	9/80 (10.1%)	8/170 (4.5%)	0.11
Total fractures (Yes/No)	36/97 (27.1%)	3/41 (6.82%)	0.01	9/80 (10.11%)	13/165 (7.30%)	0.48

^1^ Vitamin D data only available for 1 male twin

^2^ Testosterone and PTH only measured for 2 male controls

PTH: parathyroid hormone

SHBG: sex hormone binding globulin

SD =standard deviation

BMD: bone mineral density

All BMD recorded as g/cm^2^

## Results

### Characteristics of patients and controls at enrolment: Table 1

Complete data were available on 222 patients, 133(60%) males, 106(48%) Caucasians, 8 (4%) Asian and 85 (38%) African and 10% other (self-classified without further specification). HIV patients had a mean (sd) age of 45.6 (9.3) years with a median time from HIV diagnosis of 7 years [IQR: 3, 12]. Seventy-one (33%) had AIDS at diagnosis. 190(85%) were taking highly active anti-retroviral therapy (HAART), and 50(26%) were on their first combination. Therapies comprised nucleoside reverse transcriptase inhibitors 185 (99%), 146 (78%) specifically included tenofovir, and the protease inhibitor class 80(43%). Median HIV RNA level was 40(IQR: 20, 40) copies/ml, median nadir and current CD4 cell counts were 189(IQR: 75,264) and 483(IQR: 375,654), respectively. There were 222 HIV presumed uninfected singleton twin controls (all low risk for HIV infection), 44 (19.8%) were male (43 Caucasian and 1 other) and 178 (80.2%) female (all Caucasian). Apart from ethnicity, the patient and control groups were well matched for some risk factors for fragility fracture, including age and parental history of hip fracture, but there were clear differences between groups for alcohol consumption and body mass index (BMI). There were fewer males in the control group.

There was a significant disturbance in bone metabolism in HIV patients compared to controls (Table 1) for both men and women, notably reduced serum corrected calcium, elevated alkaline phosphatase, and elevated PTH (where available). HIV patients had significantly lower BMI and increased alcohol intake.

#### DXA results for Bone Mineral Density

17.6% with HIV had osteoporosis compared to 3.6% for controls, and 42.8% of the HIV group had osteopenia compared to 28.7% in controls (p<0.000). Table 2 shows the characteristics of the HIV positive patients according to age and gender. Unsurprisingly, the osteoporosis group were older compared with the groups with normal BMD or osteopenia, p<0.001. A similar proportion of younger men (<45 years) had low BMD (osteoporosis or osteopenia) as women within the same age categories (58.3%, 35/60 vs. 62.2%, 28/45, p = 0.731). For those >49 years, an increased incidence of low BMD occurred for women, with 73.1% (19/26) having low BMD compared to 63.8% men (30/47), but this was not statistically significant p=0.149). 

**Table 2:** Proportion of HIV patients (Cases) and Twins (Controls) with normal, osteopenia and osteoporosis stratified by age and gender.

	Males							Females						
Yr	Prevalence Normal		Prevalence Osteopenia		Prevalence O-Porosis			Prevalence Normal		Prevalence Osteopenia		Prevalence O-Porosis		
	HIV n *%*	Con n *%*	HIV n %	Con n %	HIV n %	Con n %	p	HIV n %	Con n %	HIV n %	Con n %	HIV n %	Con n %	p
30-34	n=8 50%	2 40	7 43.8	3 60	1 6.3	0 0	1.00	7 53.8	20 83.3	6 46.2	4 16.7	0 0	0 0	0.12
35-39	6 31.6	4 57.1	11 57.9	3 42.9	2 10.5	0 0	0.41	6 42.9	23 85.2	6 42.9	4 14.8	2 14.3	0 0	0.008
40-44	11 44	5 62.5	11 44	1 12.5	3 12	2 25	0.26	4 22.2	26 74.3	9 50	9 25.7	5 27.8	0 0	<0.001
45-49	6 23.1	4 57.1	15 57.7	3 42.9	5 19.2	0 0	0.20	8 44.4	26 70.3	7 38.9	11 29.7	3 16.7	0 0	0.02
50-54	6 26.1	3 37.5	11 47.8	3 37.5	6 26.1	2 25	0.87	4 28.6	21 72.4	7 50	7 24.1	3 21.4	1 3.4	0.01
≥55	11 45.8	3 33.3	11 45.8	4 44.4	2 8.3	2 22.2	0.57	3 25	13 50	2 16.7	12 46.2	7 58.3	1 3.8	0.001

Year: age group eg 30-34 equals 30-34 years

n: number

O-Porosis:osteoporosisCon:controls

p: p value

Table 3 shows factors associated with low BMD in the HIV patients. There were no significant differences between the groups in terms of other HIV parameters or known risk factors, including drug therapies. The osteopenic group had a lower median BMI and alkaline phosphate (ALP) than the other two groups. There were no differences between the groups for calcium, vitamin D, parathyroid hormone (PTH) or urine protein:creatinine ratio (Table 1).

**Table 3:** Factors independently associated with low bone mineral density in HIV cases.

	Crude OR (95%CI)	p-value	Adjusted OR(95 % CI)	p-value
Gender (M/F)	1.01(0.58, 1.76)	0.984	-	-
Age	1.02(0.99, 1.05)	0.284	-	-
BMI	0.93(0.88, 0.99)	0.023	0.91(0.85, 0.97)	0.006
Parent Fractured Hip (Yes/No)	0.90(0.28, 2.84)	0.853	-	-
Smoking (Yes/No)	0.90(0.48, 1.66)	0.727	-	-
Alcohol (≥3 units/day) (yes/no)	1.00(0.42, 2.38)	0.997	-	-
Vitamin D* (nmol/L)	0.81(0.51, 1.28)	0.369	-	-
Corrected Calcium (mmol/L)	1.538(0.07, 32.46)	0.782	-	-
Testosterone (nmol/L)	1.02(0.99, 1.04)	0.166	-	-
PTH* (ng/L)	0.4x10^-3^ (0.28x10^-6^, 0.65)	0.038	0.71x10^-4^ (0.12x10^-7^, 0.40)	0.03
Phosphate (mmol/L)	4.29(0.93, 19.68)	0.061	-	-
Alkaline phosphatase* (IU/L)	0.1x10^-8^ (0.23x10^-15^, 0.005)	0.008	0.51x10^-7^ (0.24x10^-14^, 1.06)	0.05
SHBG* (nmol/L)	1.72(1.00, 2.93)	0.048	1.56(0.89, 2.75)	0.12

OR=odds ratios; C= confidence interval; HAART= combination antiretroviral therapy; ALP= alkaline phosphate

* Following measures were transformed to normality previous to analysis.

PTH: parathyroid hormone

SHBG: sex hormone binding globulin

In univariate analyses, lower BMI, ALP and HAART use were associated with low BMD (Table 3). Raised serum phosphate was associated with low BMD (OR: 3.62; 95% CI: 0.84, 15.6), but did not attain statistical significance. There was a marked increase in sex hormone binding globulin (SHBG) and a reduction in free androgen index (FAI) and low free testosterone levels (P<0.001) in patients with low BMD.

After adjustments, the use of HAART was strongly associated with low BMD (aOR: 3.61; 95%CI: 1.38, 9.42). 

There was no significant difference between the BMD and the duration of HAART at the time of the DXA scan (Table 4). For those on a PI regimen, osteoporosis and osteopenia occurred in 27.8% (22/79) and 45.6% (36/79) respectively, versus 13.0% (14/108) and 47.2% (51/108) for those not on PIs (p=0.024). For those on tenofovir, the comparative figures were 19.2% (28/146) and 45.5% (65/146), versus those not on drug, 8.6% (3/35) and 45.7% (16/35), p=0.618). Patients with higher testosterone level were likely to have a low BMD (aOR 1.04; 95%CI: 1.01, 1.07) and patients with higher BMI were less likely to have a low BMD, (aOR: 0.90; 95%CI: 0.83, 0.96). Age at HIV diagnosis and ALP was associated with a slightly raised risk of osteoporosis, but this was not statistically significant.

**Table 4:** Effect of Duration of HAART on BMD.

	Duration on HAART at time of DEXA scan			total (n=181)	p (ANOVA)
	0-12 months (n=30)	13-18 months (n=12)	>18 months (n=139)		
SpineHologic BMD	0.97596667 ±0.17664878	0.93491667 ±0.11031645	0.9961223 ±0.14194909	0.98872376 ±0.1465658	0.3349
HipHologic BMD	0.9622 ±0.16673423	0.92508333 ±0.11881495	0.95506475 ±0.12778153	0.95425967 ±0.13389409	0.7139
Femoral Neck Hologic BMD	0.8306 ±0.13838716	0.78941667 ±0.10627191	0.81756115 ±0.1351834	0.81785635 ±0.13366809	0.6672

BMD measures are shown as mean± standard deviation from the mean.

All BMD recorded as g/cm^2^

All individuals that had never been on HAART (n=41) were excluded from this analysis.

BMD: bone mineral density

HAART: highly active antiretroviral therapy

#### Reported fractures

45 (20%) HIV patients, 36 male, 9 female, reported at least one previous fracture from any cause. Among controls, 16 (7%) 3 males and 13 females reported a previous fracture. Hence fracture prevalence was much higher in patients with HIV than controls, 45 (20.3%) vs. 16 (7.2%) (OR=3.27; p=0.0001).

In the HIV group, 10 year fracture risk for major osteoporotic fracture was 3.90% using BMD and 3.96% without BMD. For hip fracture risk, it was 0.46% using BMD and 0.46% without BMD. For the controls, the 10 year fracture risk for major osteoporotic fracture was 3.75% using BMD and 3.88% without BMD. For hip fracture it was 0.35% using BMD and 0.45% without BMD. 

#### Remaining Lifetime Fracture Probability *Table 5* and *Table 6*

**Table 5:** Mean Remaining Lifetime Fracture Probability of HIV+ve vs. HIV uninfected twin controls.

	Total Mean RLFP			Female Mean RLFP			Male Mean RLFP		
	Spine	Total Hip	NOF	Spine	Total Hip	NOF	Spine	Total Hip	NOF
HIV	3.20	1.27	1.26	2.85	1.62	1.54	3.44	1.04	1.08
Twins	2.12	1.39	1.77	1.96	1.61	2.05	2.80	0.47	0.64

RLFP: remaining lifetime fracture probability

NOF: neck of femur

**Table 6:** Median Remaining Lifetime Fracture Probability of HIV+ve vs. HIV uninfected twin controls.

	Total Median RLFP			Female Median RLFP			Male Median RLFP		
	Spine	Total Hip	NOF	Spine	Total Hip	NOF	Spine	Total Hip	NOF
HIV	1.98	0.65	0.55	2.01	1.12	0.83	1.95	0.54	0.46
Twins	1.44	0.85	1.17	1.38	0.94	1.39	1.74	0.25	0.34

RLFP: remaining lifetime fracture probability

NOF: neck of femur

Spine: Mean RLFPs for HIV infected vs. uninfected were: spine 3.20 vs. 2.12. p=0.0002, total hip 1.27 vs. 1.39, p=0.74, femoral neck 1.26 vs. 1.77, p=0.9957. Unadjusted odds ratios for RLFP spine was 1.22 (95%CI 1.09, 1.36), p<0.001. After adjustment for age and gender the RFLP spine was significantly higher for HIV positive patients compared to uninfected controls, with the OR remaining at 1.22 (1.07,1.40), p=0.003. Identical significance values were found when Caucasians alone were compared.

Mean RLFP for spine was greater for those on HAART, 2.4 vs. 1.5 (p=0.03), and femoral neck 0.86 vs. 0.38 (p=0.04), but there was no significant difference for total hip. These findings were unchanged when males and females were analysed separately. 

## Discussion

The increased life-expectancy for those infected with HIV mirrored the emergence of an increased incidence of age-related morbidities, such as coronary heart disease and neurocognitive disorders [2,24]. Fragility fractures may be another emerging cause of excess morbidity in HIV. We demonstrated a marked increase in the incidence of osteoporosis in HIV subjects compared to uninfected controls. A low BMI was more common in HIV, and factors adversely influencing calcium metabolism were more affected in the HIV group, notably high PTH and low vitamin D levels. We also observed a marked relative increase in previous fractures of all causality among our HIV infected subjects, although was not the prime aim of the study.

The incidence of fragility fractures may be increased in HIV for a number of reasons: there is a strong association between HIV infection and reduced BMD and this risk increases further with the addition of HAART [14]. There is also an excess in traditional risk factors for fractures in many HIV cohorts. Most HIV patients are below the age where fragility fractures are common, but there are already reports of increased rates of fractures in HIV patients. Fractures of the hip, wrist and vertebrae have been found to be more common in HIV-infected individuals[25]. The “HOPS” study mainly recruited males, and found that age-adjusted fracture rates were higher in people with HIV compared to the general US population [15]. A study among HIV infected versus uninfected male veterans in the US, found an unadjusted hazard ratio of fragility fractures of 1.32, which was reduced to 1.24 and 1.10 when adjusted for risk factors excluding and including BMI respectively [14] . Most HIV cohorts currently include few people over the age of 70, whereas the National Hip Fracture Database in the UK reports the average age of a person with hip fracture as 84 years for men and 83 for women[26]. Hence, the findings from our study might therefore herald a major problem for people with HIV as they approach their later decades of life. 

We found a three-fold increase in DXA-defined osteoporosis in HIV infected versus uninfected people, and was similar to other studies[7]. The relative risk and the absolute incidence of osteoporosis among HIV-infected subjects in the younger age groups were similar for males as for females. In contrast, for the group 55 years and older, we found that 58.3% of HIV-infected females had osteoporosis compared to only 8.3% of men (p=0.006). This may explain why a recent study comparing HIV infected versus uninfected women, showed no difference in fracture incidence between the two groups [27], as most of the participants were pre- or peri-menopausal. The terms osteoporosis and osteopenia were not intended to be used clinically for people below 40 years and we did not advise these younger patients that they had any significant disease; conversely, we did address modifiable factors identified such as smoking, alcohol, and levels of daily activity. 

An HIV-specific FRAX score would be useful for management decisions but this will require a large and long-term observational study. The 10-year risk of fracture is usually low for our HIV patients, even though many have a high number of risk factors for fragility fracture. This is because most people with HIV are currently well below the age where fragility fractures are common. Over the coming decades, fractures in people with HIV may represent an even greater cause of morbidity and mortality than for the high levels already seen among the general population[28]. The lifetime risk of fractures among our cohort indicates the potential burden of such disease in the future, and may prompt us to avert these risks by means revised screening and management. 

There was a considerably greater risk of spine fracture among male and female HIV positive recruits than for the control group. At the hip and neck of femur for HIV infected males, there was also a marked relative risk of lifetime fractures compared to controls, but for females the risks were similar at these two sites. These site specific findings may reflect the different composition of bone at the hip, which is mainly cortical (compact) in composition compared to the vertebrae which, although it has a cortical shell, is predominantly trabecular (cancellous or spongy). Spine BMD measured by quantitative CT, which estimates trabecular bone only, is usually lower and declines faster than BMD estimated by DXA (which measures trabecular and cortical bone [29]. Trabecular bone turns over more rapidly and, therefore, tends to show greater responses to agents that impede bone repair or accelerate loss. It should be emphasised that RLFP is a web-based risk calculator that was developed in a non-HIV based population, and the calculated life-time risks may turn out to be different from actual fracture rates. Determination of HIV-specific FRAX scores and the changes that we see in them over time, will enable revision of calculators of lifetime fracture risks.

The use of HAART was associated with an increased relative risk of osteoporosis, irrespective of gender. This was reflected by an increased RLFP for those on HAART, although only for the femoral neck and the spine, and not for the total hip. The initial effects of reduced BMD following HAART appear to be unrelated to the drugs used, tends to occur within the first 12 months, and the reduced BMD stabilises or reverses in the majority of individuals within approximately 18 months [30]. Some of the effects of the increased lifetime risk of fracture observed might be due to a temporary reduction in BMD in those who just commenced HAART. This is likely to be inconsequential in our study; of those subjects on HAART, the majority had been on treatment for less than 12 months, and there was no statistical difference between duration of HAART and BMD. Caution should, however, be exercised in interpreting calculators of future risk of fracture in patients on HAART, and the potential effects of reduction of BMD soon after initiation should be taken into consideration.

This study was not designed specifically to look at individual antiretroviral treatments. There was a difference between those taking protease inhibitors (PIs) or those without PIs. There was no difference between those currently taking, or not taking, a tenofovir-containing regimen. It is unlikely that an ascertainment bias would have had a significant effect on this observation because during the study period drug regimens were not altered according to fracture risk. An increased relative risk of osteopenia and osteoporosis has been associated with the use of HAART in several, but not all studies[7], and an increase in fragility fractures with protease inhibitor drugs has been observed [14]. There was an increase in fractures among those patients who remained on treatment in the Strategies for Management of Anti-Retroviral Therapy (SMART) bone sub-study, compared to those who had a treatment interruption, although there were only a few hundred in each arm and the difference did not quite reach statistical significance [31]. Although we observed that HAART was associated with a greater risk of fractures, the increase was marginal and not significant: 39/188 (20.7%) on HAART had a history of fractures compared to 5/31 (16.1%) for those naive to HAART (OR=1.57, p=0.637). 

There are advantages and limitations to our study design. Our strict approach to randomised selection of patients ensured that they were representative of the entire HIV cohort, we stratified recruitment by age, and we recruited the same number of low risk controls. Potential limitations reflected the demography of our cohorts. There was a small pool of younger controls, and fewer male controls. This was only a relative limitation as there were sufficient numbers to make meaningful comparisons for all age ranges, and the control group are much studied and are representative of the general population (14). We need to interpret the increased reported fractures with caution, as there was insufficient information to distinguish fragility fractures from traumatic fractures in many cases. It is likely that the relatively young age group, and the larger number of males in the HIV cohort, may have contributed to this relative difference in lifetime history of fractures. The large majority of the controls were Caucasian, whereas the HIV cohort was of a more mixed ethnic composition. Lipodystrophy is a frequent occurrence in people with HIV [12] and the interpretation of DXA scans might potentially be affected by this. In one study, a comparison of quantitative CT imaging of the lumbar spine with DXA, showed similar differences and trends over time amongst the different groups studied [31] . Furthermore, in our population severe body shape changes were not common, with minor changes in 22%, but there were less only 5 subjects who reported significant lipodystrophy. 

We do not know how applicable the FRAX score or the RLFP score will be for people with HIV, but we need to take heed of the increase in osteoporosis and current increase in observed fractures. Physicians should determine the risk factors that their HIV patients have for fragility fractures. If the 10 year risk is low, then the patient can be reassured but advised on how to address modifiable risk factors such as exercise, alcohol intake, diet and smoking. If the fracture risk is high, bisphosphonates should be considered as they have a similar benefit with HIV as in the general population [30–32]. Several of the risk factors for fragility fractures are shared with other common “lifestyle” diseases, for example, smoking with coronary heart disease; poor diet and low physical activity both independently linked to diabetes, coronary heart disease and malignancies; and alcohol with liver disease and malignancies. As these conditions are more frequent in people with HIV[1,33,34], this provides an additional rationale for a planned screening programme for these risk factors among the HIV population (33).
