# Correction: A Cross-Sectional Randomised Study of Fracture Risk in People with HIV Infection in the Probono 1 Study

**DOI:** 10.1371/annotation/72da3b01-3c36-4bb0-ae06-2476d8e061a6

**Published:** 2013-12-16

**Authors:** Barry S. Peters, Melissa Perry, Anthony S. Wierzbicki, Lisa E. Wolber, Glen M. Blake, Nishma Patel, Richard Hoile, Alastair Duncan, Aristotelis Tsiakalos, Ranjababu Kulasegaram, Frances M. K. Williams

Dr. Aristotelis Tsiakalos is not correctly included in the author byline. Dr. Tsiakalos should be listed as the ninth author and affiliated with Third Department of Internal Medicine, Medical School, National and Kapodistrian University of Athens, Athens, Greece. The contributions of this author are as follows: Performed the experiments. 

